# W-Band 4th Order Waveguide Filter Based on Double Layer SU8 Microfabrication

**DOI:** 10.3390/s22155604

**Published:** 2022-07-27

**Authors:** Min Liu, Qian Yang, Anxue Zhang, Cheng Guo, Juan Chen

**Affiliations:** School of Information and Communications Engineering, Xi’an Jiaotong University, Xi’an 710049, China; lm1617524795@stu.xjtu.edu.cn (M.L.); yangqiandianxin@mail.xjtu.edu.cn (Q.Y.); anxuezhang@mail.xjtu.edu.cn (A.Z.); guocheng@xjtu.edu.cn (C.G.)

**Keywords:** W-band, SU-8 photoresist, cylindrical resonators, waveguide filter, microfabrication, millimeter wave

## Abstract

This paper investigates the fabrication accuracy of the W-band SU-8 photoresist micromachined 4th order waveguide bandpass filters (BPF). The designed filter based on cylindrical resonators is excited in TM_010_ mode. It is ideally suitable for the layered SU-8 micromachining process as the height of the resonator is much smaller than one wavelength, the electromagnetic fields remain unchanged in the thickness direction. The filter is composed of three silver-coated SU-8 layers based on a double-layer overlay process. Excellent manufacturing tolerances can be controlled within 4 μm in the thickness direction, around 10 μm in double-layer stacking accuracy, and an average of 1° in vertical angle deviation. Various challenges encountered in the SU-8 process are investigated while corresponding general solutions are proposed for machining high-precision devices. The measured results show a return loss of 12.4 dB and a minimum insertion loss of 0.8 dB, which are in agreement with the simulated one. Stress and deformation analysis are also conducted to confirm the maximum pressure that the filter can withstand and maintain good transmission performance.

## 1. Introduction

With low insertion loss, high power handling, and good frequency selectivity [1], there is a growing demand for waveguide components operating in the millimeter wave (mmW) and terahertz wave (THz) frequency range, which are widely used in satellite communication systems and high-resolution imaging. High-precision waveguide devices in these frequency bands are very expensive to manufacture using conventional computer numerical control (CNC) milling techniques. To cope with the high-dimensional accuracy and surface roughness, some alternative micromachining techniques have been actively explored, such as 3D printing [2], SU-8 process [3], and Si deep reactive etching (DRIE) [4]. The latter two methods are more suitable for processing high-band devices due to the use of lithography techniques, which can pattern small features more accurately. Waveguide filters operating above 90 GHz have been extensively studied, as shown in Table 1. Table 1 shows the following results: First, devices fabricated in the SU-8 process are easier to achieve high-precision steepness than in the DRIE process. Second, The thicker the thickness of the fabricated device, the more difficult it is to control the spin coating accuracy and steepness in the thickness direction. SU-8 process is a potential micromachining technology; its advantages include good surface roughness on the sidewalls of waveguide structures, high-dimensional accuracy, being capable of building high aspect ratio structures, and reduced cost.

In this paper, a 4th order BPF has been designed using cylindrical resonators with a feature of high *Q*_u_ and fabricated using the SU-8 micromachining technique. The various challenges encountered in this fabrication process and the corresponding solutions for the precise manufacture of micro multilayer structures are put forward. To the best of our knowledge, this work is the first time to control the spin coating thickness within 4 μm in the thickness direction, double-layer overlay accuracy of about 10 μm, and 1° in vertical angle deviations on average. The main factor affecting the performance of the designed filter is the steepness, which is verified by re-simulating the filter using the measured results. The re-simulation results match well with the measured results. The influence of stress and deformation on the performance of the fabricated filter during the measurement is also analyzed in detail.

## 2. Materials and Methods

### 2.1. Design of the Filter Structure

**Figure 1:**
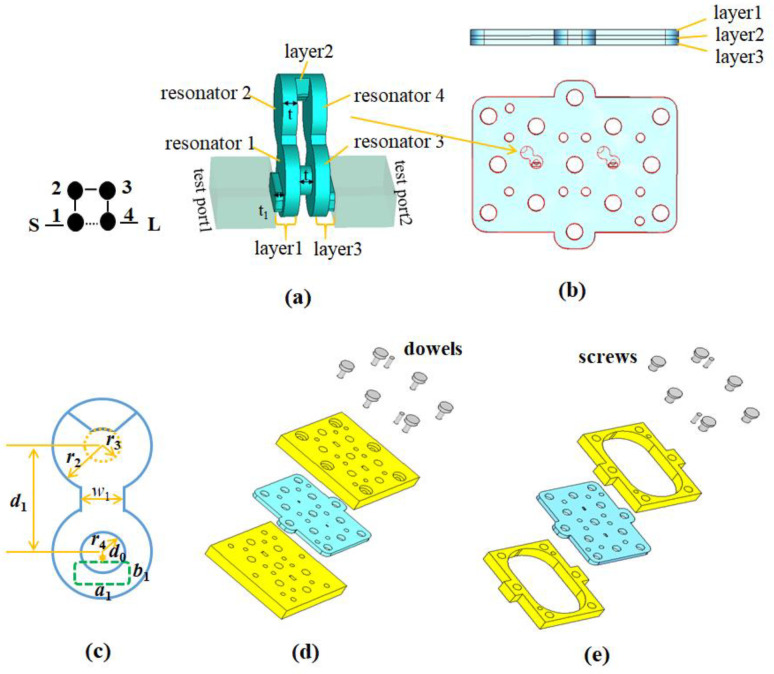
(**a**) The designed 4th BPF, (**b**) Top view and side view of the SU8 chip, (**c**) Main critical dimensions in microns are: *r*_1_ = 1233.5, *r*_2_ = 1188.9, *r*_3_ = 401.4, *r*_4_ = 498.2, *w*_1_ = 1127.2, *l*_a_ = 1631, *l*_b_ = 736.2, *d*_0_ = 91.9, *d*_1_ = 2492.6, *r*_g_ = 0.2, *t* = 500, *t*_1_ = 250, (**d**) the design of pressing block Ⅰ, and (**e**) the design of pressing block Ⅱ and an exploded view when assembled. (The orange line is the label line and not the structural part).

The BPF operating at 90 GHz with an equal ripple relative bandwidth of 4.5 GHz has been designed. The designed filter has a passband RL of 20 dB and two transmission zeros at 85.5 GHz and 94.6 GHz, respectively. Based on the specifications, the external *Q*_ext_ and the non-zero coupling coefficients between resonators are calculated to be: *Q*_ext_ = 17.64, *k*_12_ = 0.0476, *k*_23_ = 0.0436, *k*_34_ = 0.0476, and *k*_14_ = 0.0121. The structure of the presented filter is designed according to its topology, as shown in Figure 1a. The fabricated filter consists of three stacked SU-8 layers. Layer 1 or 3 contains the cavities and the input or output coupling slots and layer 2 is the coupling irises layer, as shown in Figure 1b. To make the fabricated SU-8 chips easy to be measured, two types of pressure blocks are designed, as shown in Figure 1d,e.

### 2.2. Extraction of Coupling Coefficients

**Figure 2:**
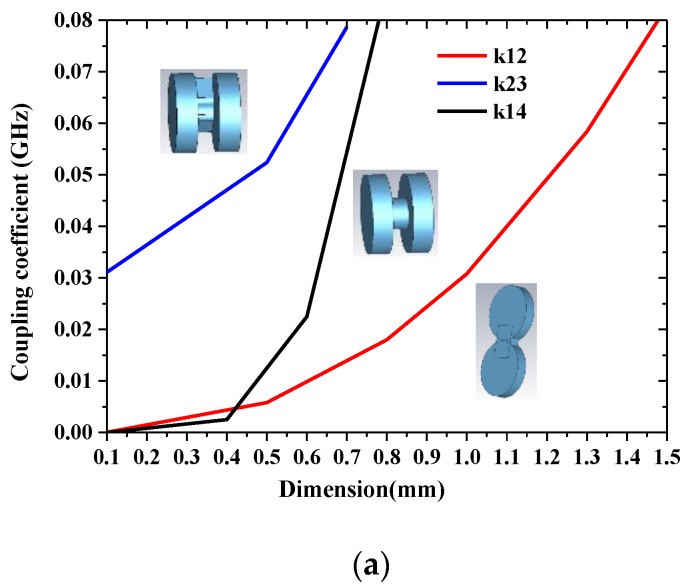

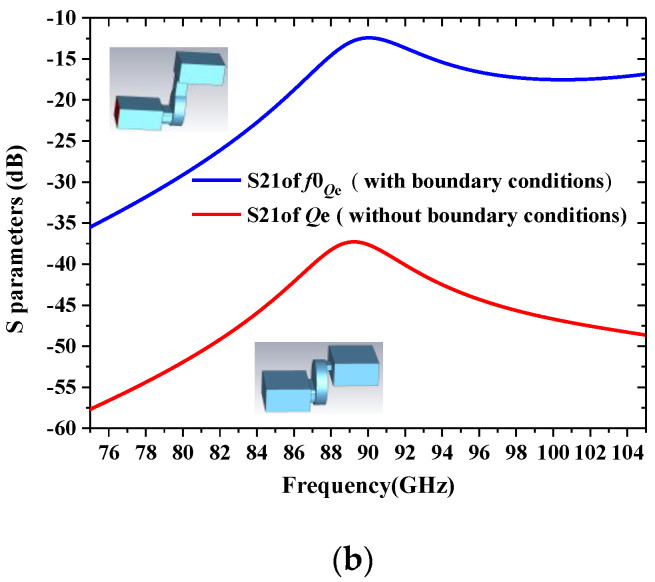
Model and coupling analysis of the cylindrical resonators: (**a**) the relation of inner-mode coupling k versus the coupling irises’ dimensions; (**b**) extract Qe’s *S*_21_ curve and model.

In the process of filter synthesis, it is necessary to convert the coupling coefficients to physical dimensions, which is described in [15]. There are many ways to extract the coupling coefficients, such as the weak coupling method, group delay method, and so on. The extraction methods of the inter-cavity coupling coefficients all use the eigenmode method. The coupling coefficient between adjacent resonators is calculated by the formula *k* = (*f*_e_^2^ − *f*_m_^2^)/(*f*_e_^2^ + *f*_m_^2^), and then the size of the coupling diaphragm between adjacent resonators is determined, as shown in Figure 2a. The external quality factor *Q*_ext_ is extracted using the weak coupling method. The boundary of resonator 1 will be affected, resulting in a shift of the resonant frequency. At this time, it is necessary to compensate for this effect by fine-tuning the size of the resonant cavity 1, as shown in Figure 2b. 

### 2.3. Analysis of the Working Mechanism of the Filter’s Operation

**Figure 3:**
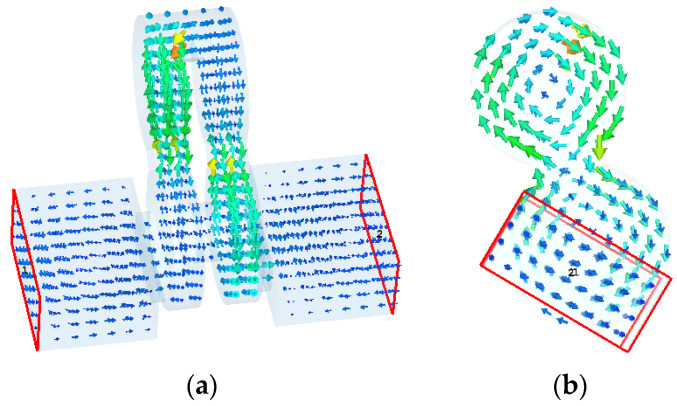
Magnetic field distribution map of the designed structure. (**a**) Front view of the designed filter (**b**) Left side view of the designed filter.

The designed filter is based on four cylindrical resonators operating in TM_010_ mode to reduce the sensitivity in the thickness direction, thereby reducing the difficulty of the processing, as shown in Figure 1a. The working mechanism of the filter can be summarized in a few steps: First, the feeding rectangular waveguide is located at the non-centrally symmetric part of resonator 1 (or 4). The arrangement ensures that the effective magnetic field of the TM_010_ is parallel to that of the feeding waveguide. Therefore, the TE_10_ mode transmitted in the fed rectangular waveguide can excite the TM_010_ mode in resonator 1; Then, resonators 1 and 2 (or resonators 3 and 4) are coupled through an inductive iris; Finally, resonators 2 and 3 are coupled via a slot. Since the whole structure is symmetric, the mode distribution on the right side of the structure is consistent with that on the right side, as shown in Figure 3.

## 3. Experimental Results

### 3.1. Fabrication of the Designed Filter

**Figure 4:**
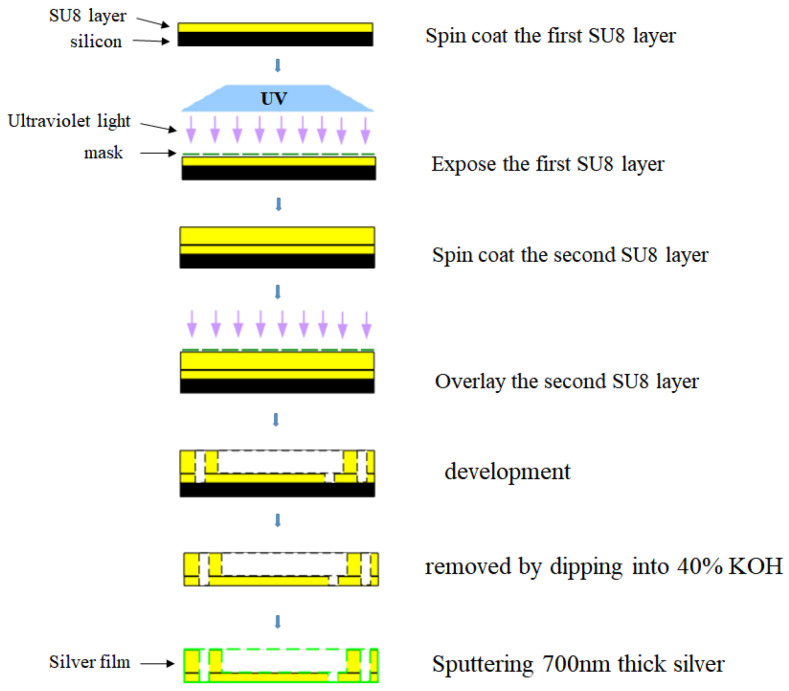
Schematic diagram of SU-8 micromachining process.

The designed filter was fabricated by the SU-8 micromachining technology. The technology mainly includes the steps of spin coating, pre-baking, exposing, post-baking, developing, removing, and sputtering. The high-precision machining process is described in detail as follows: (a) About 2.5 g of SU-8100 photoresist was spun coated on a 4-inch silicon wafer and baked it on a high-level hotplate. To more easily ensure the accuracy of the overall uniformity of the SU-8 layer, the 500 μm thick SU-8 layer is divided into two times spin coating. The SU-8 layer thickness is controlled by the weight of the photoresist spread on the silicon wafer. (b) The SU-8 photoresist was exposed to UV light at an appropriate dose. At this time, attention should be paid to the close contact between the silicon wafer and the mask to reduce diffraction and avoid processing imperfections. (c) The SU-8 layer was developed with a SU-8 developer within a suitable time. The length of development time is determined by the high aspect ratio and (or) the SU-8 layer thickness. (d) The SU-8 layer was removed from the wafer by dipping it into a 40% KOH solution. (e) All sidewalls of the SU-8 layer are deposited with 700 nm thick silver. The coated silver is at least three times the skin depth of the designed filter at 90 GHz. This will reduce the surface roughness of the device as much as possible during the metallization process, thereby reducing the insertion loss. The processing schematic diagram of the filter is shown in Figure 4. It is worth noting that during the sputtering process, if the processed SU8 chip is relatively thin (the thickness is about <400 μm), the phenomenon of SU8 chip bending will occur. Due to the large shear stress between the sputtered silver metal and the contact surface of the SU8 chip, the SU8 chip is deformed. There are two main methods to solve this problem: First, during sputtering, reduce the sputtering power in the equipment parameters so that the deposition rate of metal is slowed down, thereby reducing the possibility of device deformation due to excessive stress. Second, before sputtering, it can be fixed with tape around the edge of the SU8 chip, which can also greatly reduce the deformation of the SU8 chip.

### 3.2. Difficulties and Solutions in the Fabricating

This paper summarizes the main difficulties and corresponding solutions in the development of multi-layer platforms using SU-8 micromachining technology. They mainly include the following four aspects. 

#### 3.2.1. SU8 Micromachining Tolerance in the Thickness Direction

**Figure 5:**
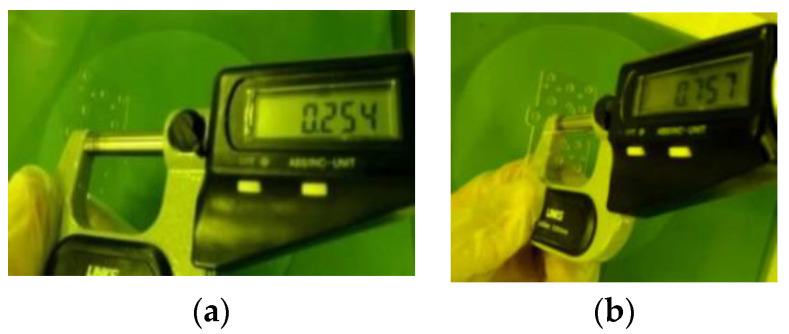
Spiral micrometer measuring SU8 chip’s thickness (**a**) the thickness of one layer and (**b**) the thickness of the SU-8 chip1 or 3.

**Figure 6:**
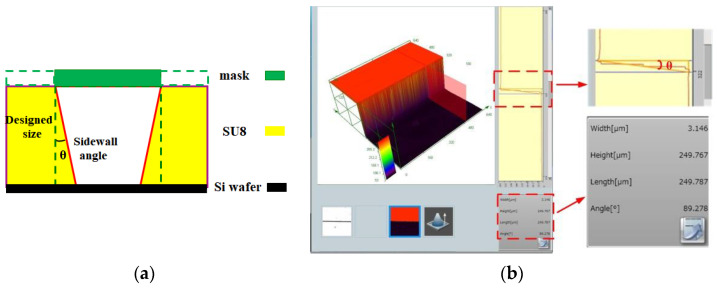
(**a**) Sidewall profile of the fabricated filter and (**b**) h = 249.7 μm, θ = 0.72° (i.e., 90–89.278°).

The precision in the thickness direction of the fabricated chip is an important factor affecting the device performance. To the best of our knowledge, the best precision can be controlled within 5 μm [9]. However, in this paper, the processing tolerance of the designed filter can be controlled within 4 μm in the thickness direction, which is measured with a screw micrometer and confocal lens, as shown in Figure 5 and Figure 6b. The measurement accuracy of the screw micrometer is 1 μm. To reduce the spin-coating precision control, the designed filter is fabricated using the same spin coating thickness or an integer multiple. The problem of controlling the SU-8 micromachining tolerance in the thickness direction can be solved by the method of thin resist compensation. 

#### 3.2.2. Underetching on Sidewalls

Steepness refers to an angle of deviation from a vertical wall. Steepness is another important factor affecting device performance in the SU-8 process. The Steepness mainly depends on the optimal exposure dose, the closeness of the contact between the silicon wafer and the mask, the wavelength of the light source, and the SU-8 photoresist thickness. A typical sidewall profile of a small cavity obtained using the SU-8 process is shown in Figure 6a. The sidewall profile exhibits an inverted isosceles trapezoid due to the fact that the actual exposure dose is larger than the optimal exposure. This reduces the effective size of the resonant cavity, which causes the self-resonant frequency of the cavity to shift to high frequencies. Deviations in the resonant frequency of a single cavity may cause severe response detuning. Therefore, the optimal exposure dose will effectively improve the steepness. In this paper, the same thickness of SU-8 photoresist is exposed to ultraviolet light with different exposure doses, and the optimal exposure dose is determined by observing the steepness. Figure 6b shows that the photoresist thickness of 249.7 μm corresponds to a steepness of 0.72° as measured by a confocal lens. Another method for determining the steepness is to calculate it more accurately by the tangent formula mentioned in Section 5 of this paper.

**Figure 7:**
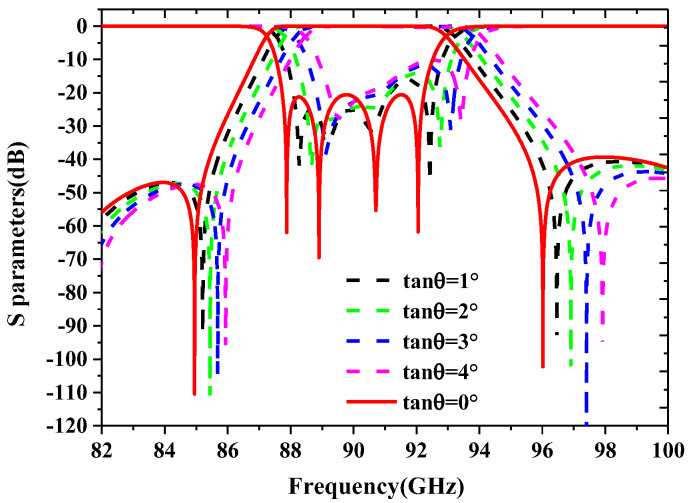
The sensitivity analysis of the steepness of the designed filter is based on simulations.

Since the main factor affecting the frequency offset and bandwidth variation of the device is caused by the steepness of the resonators and the coupling irises between them, the sensitivity of their steepness is analyzed in this paper, as shown in Figure 7. It shows a maximum steepness tolerance of 4° that can be tolerated for performance while the device is guaranteed to operate properly.

#### 3.2.3. Overlay Accuracy of Multilayer Structures

**Figure 8:**
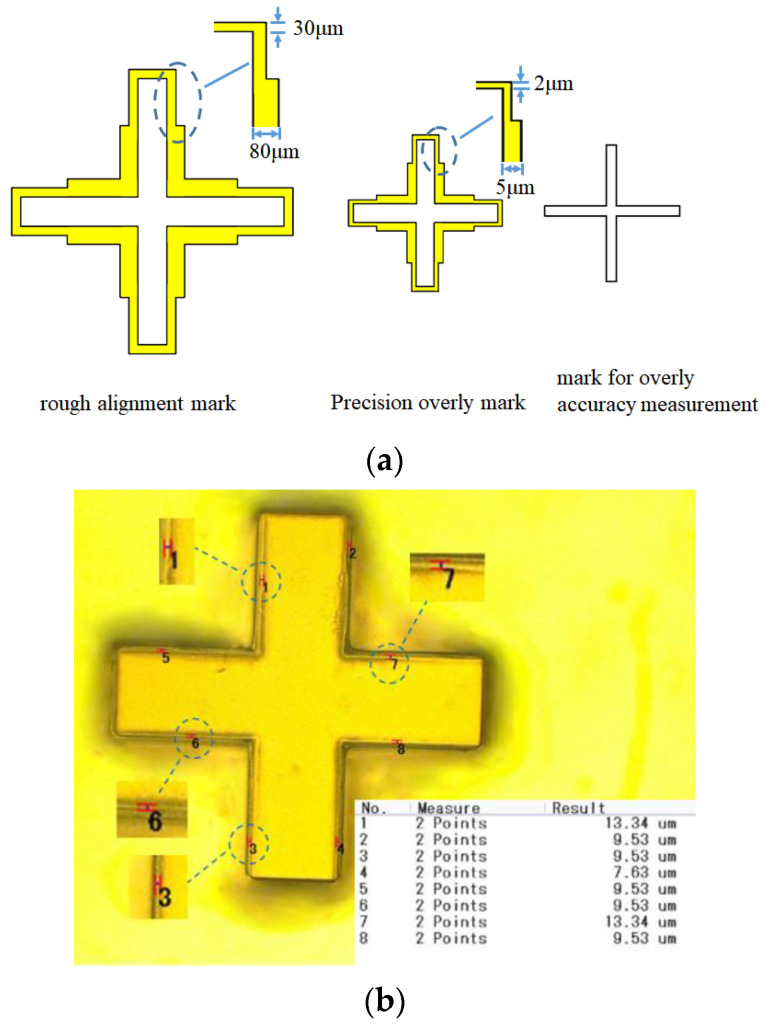
Multi-layer overlay marking (**a**) Design of the engraved marks and measured marks for masks (**b**) Precision measurement of overlays, and the maximum tolerance is 13.34 μm.

To reduce the tolerance introduced by the alignment of multilayer SU-8 chips, this paper adopts a two-layer overlay method to reduce the number of SU-8 chips during assembly. Alignment difficulty during overlay is reduced by using a method in which the overlay marks of the first layer are designed to be smaller than those of the second layer. However, the size of the measurement marks on the two layers is the same to facilitate the measurement of the overlay accuracy, as shown in Figure 8a. Figure 8b shows that the engraving accuracy of the two-layer structure in this article is about 10 μm.

#### 3.2.4. Chip-to-Chip Alignment

**Figure 9:**
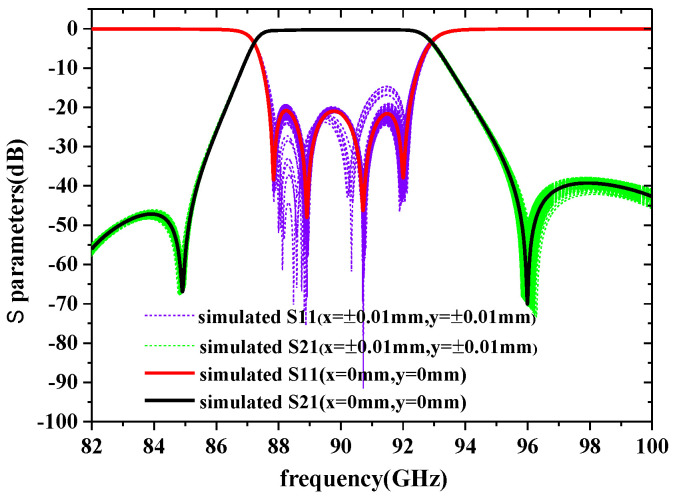
Sensitivity analysis of the designed filter based on simulations, x represents a move in the X direction, and y represents a move in the Y direction.

The alignment between the SU-8 chips also has a certain impact on the entire filter’s performance. Misalignment will cause a detuning response or excite higher-order harmonics. The sensitivity analysis of the designed filter is shown in Figure 9. It shows how the misalignment of only the coupling irises layer of about 10 μm in the horizontal and vertical directions with respect to the SU-8 chip1 or 3 affects the entire filter’s response. To improve the alignment accuracy between SU-8 chips, the alignment pin holes in the waveguide flange we designed to adopt the combination of circular and elliptical alignment holes in [16], and the alignment rate of this method can reach 99.5%. The designed flange contains 4 waveguide flange alignment holes, which is described in the IEEE1785.2a standard [17].

## 4. Measurement Results and Discussion

### 4.1. Design of the Press Blocks and Construction of Measurement Platform

**Figure 10:**
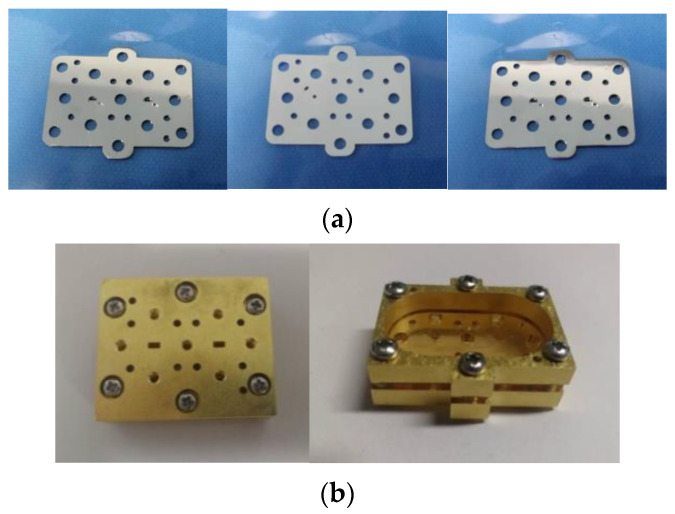
Structure diagram of processing filter. (**a**) Filter composed of 3 layers of SU-8 and (**b**) a picture of the measuring platform with pressure block Ⅰ on the left and with pressure block Ⅱ on the right.

**Figure 11:**
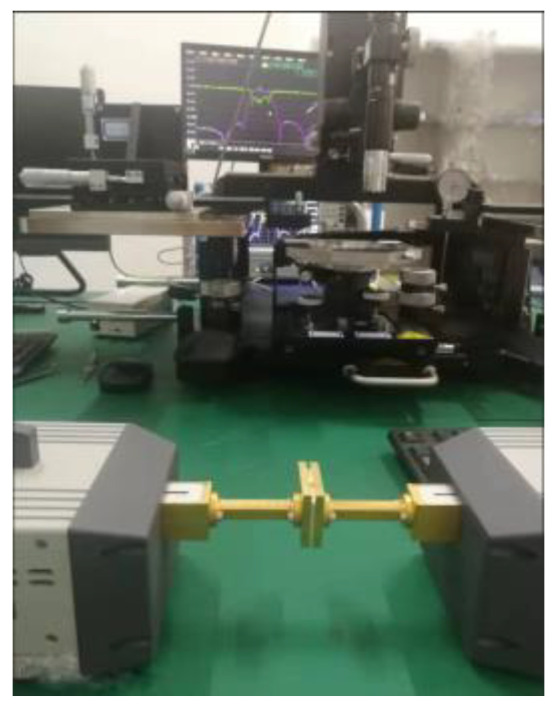
Photograph of the measurement setup.

The filter mentioned in this article is designed to be inserted into the center of a standard WR-10 flange to reduce insertion loss due to leaky waves. To eliminate the possibility of gaps between the SU-8 layers during the measurements, two kinds of pressure blocks are designed to help the measurement, as shown in Figure 10b. The difference between the two kinds is that pressure block Ⅰ has a standard WR-10 straight waveguide with a length of 15 mm, and the fabricated filter needs to be measured through the waveguide transmission line of the pressure block. While the pressure block Ⅱ can be directly connected to the waveguide port of the test equipment to the feeding rectangular waveguide. Pin holes and screw holes on the pressure block can ensure good alignment and tight contact between the SU-8 chip and the pressure block, as shown in Figure 10b. The measurement setup is shown in Figure 11, showing the SU-8 chips sandwiched between the two types of press blocks.

### 4.2. Analysis of Measurement Results

**Figure 12:**
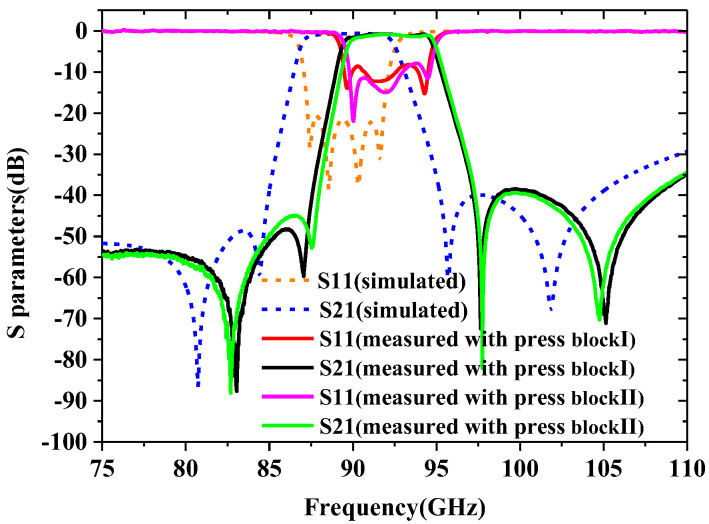
Simulation and measurement results of the designed filter over the whole *W*-band with two kinds of press block.

The S-parameter measurement and simulation results under the two different press blocks are shown in Figure 12. The measured results of clamping the SU-8 chip with the pressure blockⅠshow that the center frequency is 92 GHz, the equal ripple bandwidth is 5.5 GHz, the minimum insertion loss is 0.8 dB, and the return loss is better than 12.4 dB. The measured results of clamping the SU-8 chip with the pressure block Ⅱshow that the center frequency is 92.25 GHz, the equal ripple bandwidth is 5.5 GHz, the minimum insertion loss is 0.9 dB at this center frequency, and the return losses are better than 11.7 dB across the whole passband. The reason for the higher center frequency of the passband and the narrower FBW is that manufacturing imperfections are not considered in the design. The measured insertion loss of the pressure block Ⅰ is 0.1 dB smaller than that of the pressure block Ⅱ. This is due to the fact that the SU-8 chip with pressure block Ⅱ has a large gap between the layers which introduces additional losses. The measured insertion loss using the two kinds of pressure block structures are higher than the theoretical insertion loss value of 0.27 dB using the conductivity of silver. The reasons for this small difference in insertion loss may be attributed to the following aspects: (i) Small calibration errors. (ii) Imperfect contact between adjacent layers makes the return loss worse, thereby reducing the insertion loss. (iii) The imperfect surface roughness can degrade the effective conductivity to 2/3 and cause another loss. (iv) The type and thickness of surface metallization of the waveguide’s sidewall have an effect on the insertion loss [18].

### 4.3. Modify the Model of the Designed Filter

**Figure 13:**
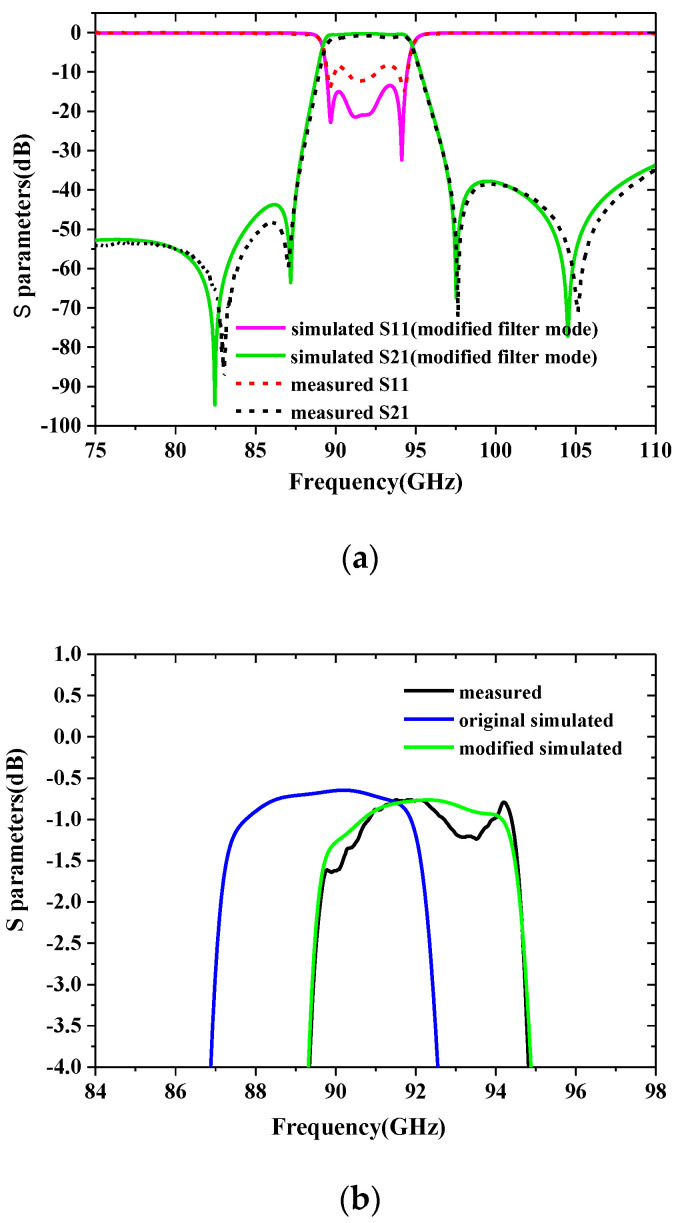
Measurement results and simulation results using metal block Ⅰ. (**a**) Responses over the whole *W*-band. (**b**) Expanded view of *S*_21_ over the passband.

The physical dimensions for the SU8 fabricated filter are measured using a confocal lens, as shown in Table 2. It shows that the measured dimensions are about 23.7 μm smaller than the designed size on average in the two-dimensional plane directions of *x* and *y*. The average sidewall steepness calculated by the tangent formula is 1°. The exposure dose used in this paper is slightly larger than the optimal exposure dose. This causes a part of the UV light to be reflected so that the SU-8 resist is exposed again. Therefore, the steepness of the devices fabricated can be further addressed by reducing exposure doses. The various tolerances mentioned above should be considered in the model design to ensure that the re-simulated and measured results with the pressure block Ⅰ match perfectly, as shown in Figure 13. Compared with the originally designed structure, the modified model uses a scaling factor of 1.0065, which is attributed to the fact that the actual size of the machined filter is smaller than the design size. Through the above analysis, the sidewall is the main parameter that affects the performance of the device. There are two methods to improve the test results: First, the tolerance caused by the steepness can be compensated by the change of the size during the design. Second, a method without standing waves in the direction of the steepness is adopted so that the frequency of the resonator can be adjusted to the steepness of the sidewall of the resonator, thereby reducing the sensitivity of the device performance to the steepness.

### 4.4. Stress and Deformation Analysis of the Fabricated SU8 Chips during Measurement

**Figure 14:**
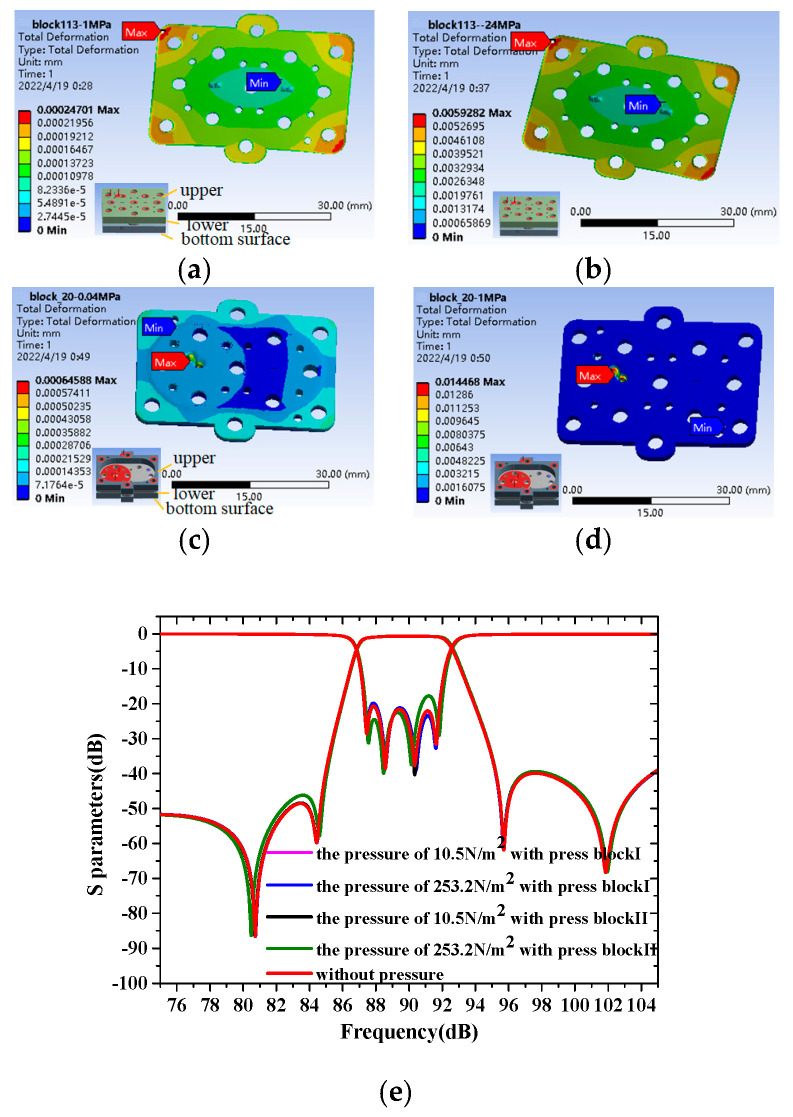
Finite element analysis of stress and deformation of the SU-8 chips with applied forces of 0.1 MPa (**a**,**c**) and 0.04 MPa (**b**,**d**) under the action conditions of pressure block 1 (**a**,**b**) and pressure block 2 (**c**,**d**). (**e**) The effects of deformation under the stress of the above four cases.

Waveguide transmission lines and many screw holes and alignment pin holes are added to the SU-8 chip, which makes the SU-8 chip discontinuous, resulting in the largest local stress. To ensure that the applied pressure does not exceed the −15 μm maximum limit of the corresponding deformation amount, it is necessary to analyze the stress and deformation finite element using ANSYS. The most deformed part of the SU-8 chip is mainly concentrated in the filter at the center of the chip. In the actual measurement, the SU-8 chip needs to be measured with the help of two kinds of pressing blocks. Therefore, the stress on the SU-8 chip is mainly concentrated in the z-direction, which has been verified in ANSYS. The physical properties of SU-8 polymer are as follows: Tensile strength of 60 Mpa, Young’s Modulus of 2.0 Gpa, Elastic Modulus of 4.6 Gpa, and Poisson’s ratio of 0.25. The bottom surface of the lower pressure block of pressure block Ⅰ is set as a fixed support, and the top screw hole of the upper-pressure block exerts a pressure of 10.55 N/m^2^ on the area of the size of the nut. The deformation of the fabricated filter is 0.11 μm, as shown in Figure 14a. Under the pressure of 10.55 N/m^2^, the deformation of the designed filter under the action of the pressing block Ⅱ is 0.43 μm, as shown in Figure 14c. Under the action of pressing block Ⅱ, the maximum stress that the SU-8 chip can withstand is about 253 N/m^2^, so the maximum deformation of the filter is controlled within 15 μm, as shown in Figure 14d. The same pressure of 253 N/m^2^ was applied to the SU-8 chip under the action of pressing block Ⅰ, and the deformation of the designed filter is 2.6 μm, as shown in Figure 14b. The above analysis shows that the cavity filter fabricated by the SU-8 process using the pressing blocks-assisted measurement has good mechanical properties for mmW applications. Figure 1d shows the effects of deformation on filter performance under the stress of the above four cases. It illustrates that there is less impact on the overall filter performance in the deformation of 15 μm.

## 5. Conclusions

The manufacturing accuracy of the 90 GHz BPF using the cylindrical resonator designed by the multilayer chip platform has been proven. The machining accuracy in the thickness direction of the cavity can reach 4 μm. The precision of multi-layer engraving can reach about 10 μm. The angle of the cavity side-wall deviating from the vertical direction can be controlled at 1° on average. The measured *S*_11_ with press block I was better than 12.4 dB. By bringing the size of the actually fabricated filter into the simulation model and re-simulation, the re-simulation results show that the main factor affecting the filter performance is the device’s steepness. This is further confirmed that the solution to the challenges encountered in the machining process proposed in this paper is feasible and has reference value for the improvement of the precision of the SU-8 micromachining process.

## Figures and Tables

**Table 1:** Recently published waveguide filter operating at frequencies above 90 GHz.

Ref.	*f*_0_, GHz	Resonators	Technology	Thickness, μm	Thickness Error, μm	Steepness
[5]	220–321	Rectangular, TE_101_	SU-8	432	about 10–15	*
[6]	293.2	Rectangular, TE_101_	SU-8	432	about 10–15	*
[7]	100	Rectangular, TE_101_	SU-8	635	about 15	max of 1°
[8]	671	Rectangular, TE_101_	SU-8	191 **	*	0.6°
[9]	100	Rectangular, TE_201_	SU-8	1270	5	1.3°
[10]	100	Rectangular, TE_102_ TE_301_	SU-8	1270	*	1–2°
[11]	400	elliptic cavity, TM_110_	DRIE	280	5	1.46°
[12]	298.6	Rectangular, TE_101_	SU-8	432	about 10–15	*
[13]	450	Rectangular, TE_101_	DRIE	30/275	equipment accuracy	*
[14]	270	Elliptic, quasi-TM_110_	DRIE	30/275	equipment accuracy	about 3°
This work	90	Cylindrical, TM_010_	SU-8	250/500	4	1°

* It is not mentioned in the article. ** Multiple single-layer SU-8 chips.

**Table 2:** Designed and measured physical dimensions for the SU-8 fabricated filter (unit: µm).

Design *	*r*_1_	*r*_2_	*r*_3_	*r*_4_	*w*_1_	*l*_a_	*l*_b_
Value	1233.5	1188.9	401.4	498.2	1127.2	1631	736.2
Measured *	*r*_1_	*r*_2_	*r*_3_	*r*_4_	*w*_1_	*l*_a_	*l*_b_
Value	1206.8	1153.8	421	495.2	1106.2	1592.5	721
Δ*l*	26.7	35.1	19.6	3	21	38.5	15.2
Steepness	1.24	1.63	0.91	0.14	0.98	1.79	0.7

* parameter. Δ*l* = designed dimensions—measured dimensions.
